# New records of reptiles from Kim Bang Proposed Species and Habitat Conservation Area, Vietnam

**DOI:** 10.3897/BDJ.14.e173158

**Published:** 2026-01-26

**Authors:** Anh Van Pham, Cuong The Pham, Tien Quang Phan, Minh Duc Le, Linh Thuy Ha, Vinh Dinh Ninh, Truong Quang Nguyen

**Affiliations:** 1 Faculty of Environmental Sciences, University of Science, Vietnam National University, Hanoi, 334 Nguyen Trai Road, Hanoi 11416, Vietnam Faculty of Environmental Sciences, University of Science, Vietnam National University, Hanoi, 334 Nguyen Trai Road Hanoi 11416 Vietnam; 2 Graduate University of Science and Technology, Vietnam Academy of Science and Technology, 18 Hoang Quoc Viet Road, Hanoi 10072, Vietnam Graduate University of Science and Technology, Vietnam Academy of Science and Technology, 18 Hoang Quoc Viet Road Hanoi 10072 Vietnam; 3 Institute of Biology, Vietnam Academy of Science and Technology, 18 Hoang Quoc Viet Road, Hanoi 10072, Vietnam Institute of Biology, Vietnam Academy of Science and Technology, 18 Hoang Quoc Viet Road Hanoi 10072 Vietnam; 4 Central Institute for Natural Resources and Environmental Studies, Vietnam National University, Hanoi, 19 Le Thanh Tong, Hanoi, Vietnam, Hanoi 11021, Vietnam Central Institute for Natural Resources and Environmental Studies, Vietnam National University, Hanoi, 19 Le Thanh Tong, Hanoi, Vietnam Hanoi 11021 Vietnam; 5 Department of Herpetology, American Museum of Natural History, Central Park West at 79th Street, New York 10024, USA, United States of America Department of Herpetology, American Museum of Natural History, Central Park West at 79th Street New York 10024, USA United States of America

**Keywords:** sistribution, lizard, morphology, Ninh Binh Province, snakes

## Abstract

**Background:**

Kim Bang Proposed Species and Habitat Conservation Area (SHCA) is located in Ninh Binh Province, northern Vietnam, with an area of 3,400 hectares of natural forest. The terrain of the proposed SHCA is characterised by limestone karst formation and a narrow valley. However, the reptile fauna of Kim Bang SHCA is still poorly studied, with only 17 species of reptiles recorded from this area so far. This study aimed to provide novel data on the distribution of reptiles with their taxonomy and natural history from the Kim Bang Proposed Species and Habitat Conservation Area of Vietnam.

**New information:**

As a result of our field surveys in 2025, we herein report ten species of reptiles for the first time from Kim Bang SHCA, namely *Chrysopelea ornata* (Shaw, 1802); *Takydromus sexlineatus* Daudin, 1802; *Ahaetulla prasina* (Boie, 1827); *Boiga multomaculata* (Boie, 1827); *Hemidactylus garnotii* Duméril & Bibron, 1836; *Protobothrops mucrosquamatus* (Cantor, 1839); *Pareas margaritophorus* (Jan, 1866); *Gekko palmatus* Boulenger, 1907; *Ptyas multicincta* (Roux, 1907); and *Tropidophorus hainanus* Smith, 1923 with morphological data. Our findings bring the species number of reptiles known from this area to 27. In addition, we provide natural history notes of the aforementioned species.

## Introduction

In Southeast Asia, karsts cover an area of around 400,000 km^2^ (Day and Urich 2000 and Clements et al. 2006). Karsts in this region, which are most extensive in Indonesia, Thailand and Vietnam and these are known to contain high levels of endemism (Clements et al. 2006). Many of these outcrops, which have historically been spared from agricultural development because of their rugged terrain, may function as biodiversity reservoirs (Clements et al. 2006) as well as serving as natural laboratories for biogeo-graphical, ecological, evolutionary and taxonomic research (Schilthuizen et al. 2005).

Limestone karst forests in the northern region are recognised as a biodiversity centre in Vietnam with a high level of species richness and local endemism (Sterling et al. 2006). In the past five years, 13 new species of reptiles have been discovered from limestone karst habitats in northern Vietnam, comprising eight species of the family Gekkonidae (Do et al. 2020, Nguyen et al. 2020, Le et al. 2021, Luu et al. 2023, Luu et al. 2024, Tran et al. 2024 and Luu et al. 2025), one species of the family Homalopsidae (Bernstein et al. 2022), two species of the family Scincidae (Pham et al. 2024 and Bragin et al. 2025) and two species of the family Xenodermidae (Luu et al. 2020 and Ha et al. 2022).

Kim Bang SHCA was included in the National Biodiversity Conservation Plan for the period 2021-2030 with an area of 3,400 hectares of limestone karst forest in Ninh Binh Province, Vietnam (The Government of Vietnam 2024). In the herpetofaunal list of Kim Bang, Do et al. (2022) recorded 17 species of reptiles, comprising two species of the family Agamidae (*Acanthosaura lepidogaster*, *Calotes emma*), three species of the family Gekkonidae (*Cyrtodactylus soni*, *Gekko reevesii*, *Hemidactylus frenatus*), one species of the family Pythonidae (*Python molurus*), five species of the family Colubridae (*Coelognathus radiatus*, *Elaphe moellendorffi*, *Lycodon futsingensis*, *Oligodon chinensis*), one species of the family Homalopsidae (*Hypsiscopus plumbea*), three species of the family Elapidae (*Bungarus candidus*, *B. fasciatus*, *Naja atra*), one species of the family Pareatidae (*Pareas hamptoni*) and one species of the family Geoemydidae (*Cuora mouhotii*) (Do et al. 2022).

The purpose of this study is to provide novel data on the distribution of ten reptile species with their taxonomy and natural history, based on our recent field survey work in the Kim Bang SHCA between April and August 2025.

## Materials and methods

**Sampling**

**Figure 1.**
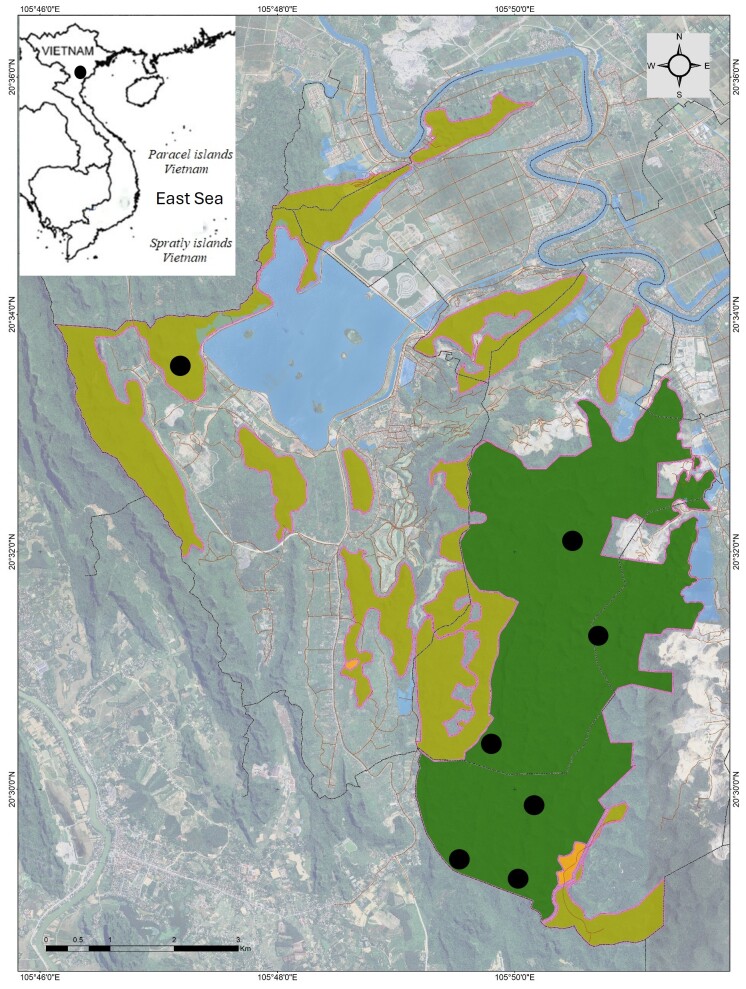
Map showing sampling sites (black circle) within Kim Bang Species and Habitat Conservation Area in Ninh Binh Province, Vietnam

Field surveys were conducted in Kim Bang SHCA from 21 to 29 April and 21 to 31 July 2025 by T.Q. Nguyen, C.T. Pham, A.V. Pham and T.Q. Phan (Fig. 1). The coordinates (WGS 84) and elevations were determined by using the GPS Garmin 62SX. Specimens were collected by hand or snake hook between 9:00 h and 23:00 h. Specimens were euthanised in a closed vessel with a piece of cotton wool containing ethyl acetate (Simmons 2002), fixed in 80% ethanol for five hours and then transferred to 70% ethanol for permanent storage. Voucher specimens were subsequently deposited in the collections of the Institute of Biology (IB) and Faculty of Environmental Sciences, University of Science (HUS), Vietnam National University, Hanoi (VNU), Vietnam.

**Morphological characters**

Measurements were taken with a digital caliper to the nearest 0.1 mm. Terminology of morphological characters followed Nguyen et al. (2010) for skinks, Nguyen et al. (2013) for geckos and Pham et al. (2020) for snakes: SVL: snout-vent length, from tip of snout to anterior margin of cloaca; TaL: tail length, from posterior margin of cloaca to tip of tail. Bilateral scale counts were given as left/right.

Gecko: nasals, supralabials, infralabials, interorbitals, preorbitals, dorsal tubercle rows at mid-body, granules surrounding dorsal tubercles, scales in a line from mental to the front of cloacal slit, scale rows at mid-body, ventral scale rows at mid-body, subdigital lamellae under fourth finger, subdigital lamellae under fourth toe, precloacal pores, postcloacal tubercles.

Skink: nuchals, supralabial scales, infralabial scales, paravertebral scales, the number mid-body scales rows, ventral scales, scale rows at position of tenth subcaudal on tail including subcaudal, lamellae beneath the fourth finger and fourth toe.

Snake: supralabials, infralabials, temporals, ventral scales, anterior dorsal scale rows at neck, mid-body dorsal scale row, posterior dorsal scale rows, subcaudal scales.

## Taxon treatments

### Gekko palmatus

Boulenger, 1907

BC742FC4-BD51-511F-A2FE-50EFA8F3FB59

#### Materials

- **Type status:** Other material. **Occurrence:** catalogNumber: IB R.6496 (Field number TC-HN 2025.47); individualCount: 1; sex: male; lifeStage: adult; occurrenceID: 3DCC2F33-2478-5119-8D5A-E4CE8FBB2772; **Taxon:** scientificNameID: *Gekko palmatus*; scientificName: *Gekko palmatus*; class: Reptilia; order: Squamata; family: Gekkonidae; genus: Gekko; specificEpithet: *palmatus*; scientificNameAuthorship: Boulenger, 1907; **Location:** country: Vietnam; countryCode: VN; stateProvince: Ninh Binh; county: Kim Bang; municipality: Kim Bang; locality: Kim Bang SHCA; verbatimElevation: 250; verbatimLatitude: 20°31204"N; verbatimLongitude: 105°50583"E; verbatimCoordinateSystem: WGS84; **Event:** eventDate: 23 April 2025; eventRemarks: collected by Nguyen QT, Pham TC, Phan QT, and Pham VA; **Record Level:** language: en; collectionCode: Reptilia; basisOfRecord: Preserved Specimen

#### Description

Morphological characters of the specimen from Kim Bang were consistent with those in the descriptions by Hecht et al. (2013) and Nguyen et al. (2013): SVL 66.9 mm, tail regenerated, TaL 48.6 mm; supralabials 10; interorbitals 30; upper ciliary scales 2 times as large as medial snout scales, 25–29 in number, 4 spinous tubercles posteriorly; a skin fold running from the last supralabial, backwards about half way to tympanum; ear opening oblique, oval, with a skin fold above; temporal region with several tubercles above tympanum; infralabials 9; dorsal tubercle 2 or 3 times as large as adjoining dorsal scales, round to oval, convex, smooth, surrounded by 9 dorsal scales, in 8 longitudinal rows at mid-body; lateral fold present, without tubercles; ventrals between lateral folds 37; scales around mid-body in 144 rows; ventral scales in a line between mental and cloacal slit 186; enlarged femoral scales absent; fingers and toes webbed basally; subdigital lamellae under first finger 10, under fourth finger 13, under first toe 9, under fourth toe 13; precloacal pores 23, in a continuous row; enlarged scales posterior to precloacal pores in 3 rows; postcloacal tubercle 1/1, blunt; tail thickened at base, with some tubercles on dorsal surface of tail base; subcaudals flat, enlarged.

**Figure 2.**
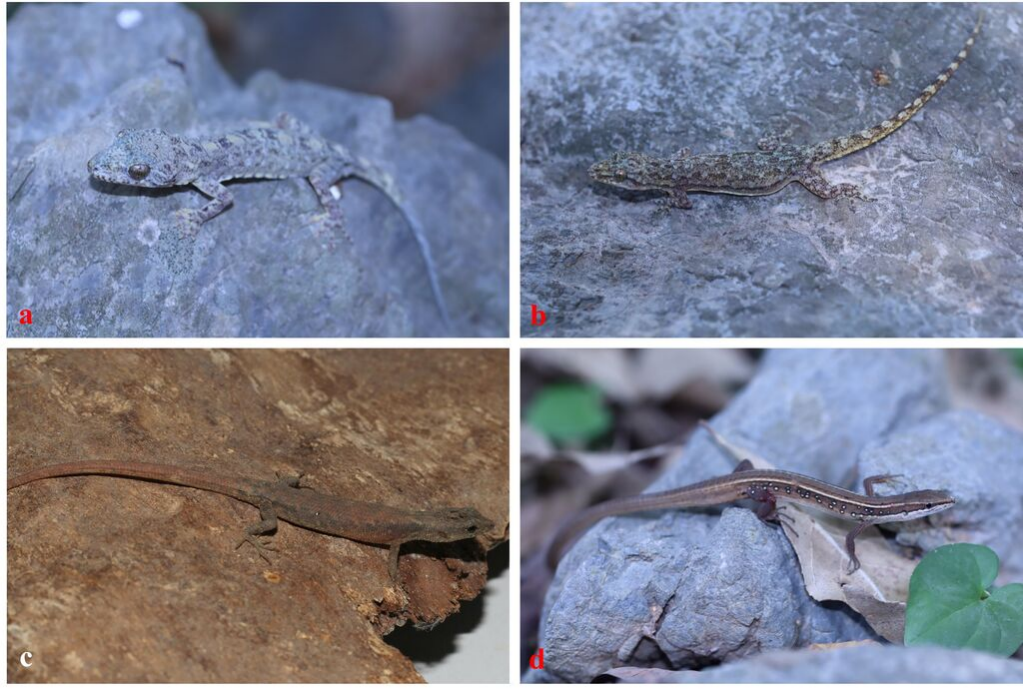
Lizard species recorded in Kim Bang Proposed Species and Habitat Conservation Area, Vietnam: **(a)** *Gekko palmatus*; **(b)** *Hemidactylus garnotii*; **(c)** *Tropidophorus hainanus*; **(d)** *Takydromus sexlineatus*.

**Colouration in life.** Dorsal surface of head and body grey with a small light blotch on neck and five larger blotches between shoulder and sacrum; flanks with some small light spots between limb insertions; limbs with light spots; dorsal tail with three light bands; throat, venter and precloacal region yellowish-cream with dark dots (Fig. 2a).

#### Distribution

In Vietnam, this species is currently known only from northern Vietnam: Thai Nguyen, Bac Ninh, Lao Cai, Son La, Phu Tho, Lang Son, Quang Ninh and Quang Binh Provinces (Nguyen et al. 2013 and Pham et al. 2018).

#### Ecology

The specimen was found at 20:30 h, on limestone karst outcrops, 1 m above the ground. The surrounding habitat is a secondary forest of small hardwood and shrubs.

### Hemidactylus garnotii

Duméril & Bibron, 1836

1BF13D14-BF0C-5CEF-8B42-E0F566D5AD5E

#### Materials

- **Type status:** Other material. **Occurrence:** catalogNumber: IB R.6497 (Field number TC-HN 2025.6); individualCount: 1; sex: female; lifeStage: adult; occurrenceID: 12C16AD7-9D7B-501F-BA0A-1738608C759E; **Taxon:** scientificNameID: *Hemidactylus garnotii*; scientificName: *Hemidactylus garnotii*; class: Reptilia; order: Squamata; family: Gekkonidae; genus: Hemidactylus; specificEpithet: *garnotii*; scientificNameAuthorship: Duméril & Bibron, 1936; **Location:** country: Vietnam; countryCode: VN; stateProvince: Ninh Binh; county: Kim Bang; municipality: Kim Bang; locality: Kim Bang SHCA; verbatimElevation: 50; verbatimLatitude: 20°31308"N; verbatimLongitude: 105°48214"E; verbatimCoordinateSystem: WGS84; **Event:** eventDate: 21 April 2025; eventRemarks: collected by Nguyen QT, Pham TC, Phan QT, and Pham VA; **Record Level:** language: en; collectionCode: Reptilia; basisOfRecord: Preserved Specimen

#### Description

Morphological characters of the specimen from Kim Bang resemble those in the descriptions by Smith (1935), Zug et al. (2007) and Pham et al. (2018): SVL 58.0 mm, TaL 69.8 mm; head moderately large and broad; rostral large, as high as broad, rectangular with mid-dorsal cleft; nares bordered by rostral, a large supranasal, two postnasals and first supralabial; supranasals separated from each other by a small scale; supralabials 10; infralabials 8; mental triangular, two pairs of chin-shields; back without enlarged tubercles; ventral scales slightly enlarged, from base of neck to pelvic area; subdigital lamellae on pad extending to base of digit and slightly on to palm/sole: 9 on fourth finger, distalmost lamella undivided, subsequent 4–8 divided; 11 on fourth toe, distalmost lamella undivided, subsequent 5–10 divided; femoral and precloacal-pores absent; tail mid-ventrally with rectangular, slightly overlapping, smooth-surfaced plates from vent to at least mid-length, reducing in size to small elongate conical scales on ventrolateral edges; tail depressed, segmented, each segment 7 scales long; ventrolaterally sawblade like with three conical spine scales in each segment, posteriormost one at edge of each segment double the size of preceding three.

**Colouration in life.** Dorsal surface of head, body, limbs and tail greyish with small white spots; venter immaculate cream to light yellow (Fig. 2b).

#### Distribution

In Vietnam, this species has been recorded from Son La, Thai Nguyen and Hai Phong Provinces in the north, southwards to An Giang and Ca Mau Provinces (Pham et al. 2018). Elsewhere, the species is known from India, Bangladesh, Nepal, Bhutan, Thailand, Myanmar, Cambodia, China, Taiwan, the Philippines, Indonesia, Malaysia, New Guinea, New Caledonia, Loyalty Islands, Polynesia, Fiji, Western Samoa, Vanuatu, Cook Islands and Tonga (Uetz et al. 2025).

#### Ecology

The specimen was found at 20:00 h, on a tree, approximately 1.5 m above the ground. The surrounding habitat is secondary forest of small hardwoods mixed with shrubs and vines.

### Tropidophorus hainanus

Smith, 1923

23A8C07A-41B8-5C18-B49D-74197C9B8F48

#### Materials

- **Type status:** Other material. **Occurrence:** catalogNumber: IB R.6498 (Field number TC-HN2025.203); individualCount: 1; sex: female; lifeStage: adult; occurrenceID: C52A3AD8-ED50-5512-BBAC-1DDC783669D9; **Taxon:** scientificNameID: *Tropidophorus hainanus*; scientificName: *Tropidophorus hainanus*; class: Reptilia; order: Squamata; family: Scincidae; genus: Tropidophorus; specificEpithet: *hainanus*; scientificNameAuthorship: Smith, 1923; **Location:** country: Vietnam; countryCode: VN; stateProvince: Ninh Binh; county: Kim Bang; municipality: Kim Bang; locality: Kim Bang SHCA; verbatimElevation: 290; verbatimLatitude: 20°31496"N; verbatimLongitude: 105°50131"E; verbatimCoordinateSystem: WGS84; **Event:** eventDate: 26 April 2025; eventRemarks: collected by Nguyen QT, Pham TC, Phan QT, and Pham VA; **Record Level:** language: en; collectionCode: Reptilia; basisOfRecord: Preserved Specimen

#### Description

Morphological characters of the specimen from Kim Bang were similar to those in the descriptions by Nguyen et al. (2010) and Hecht et al. (2013): SVL: 37.2 mm, TaL: 54.2 mm; upper head scales strongly striated; supranasal absent; frontonasal undivided; prefrontals separated from each other; frontoparietals in contact with each other medially and with 3 posterior supraoculars; interparietal with a small transparent spot; parietals large, separated anteriorly by a small scale, posteriorly bordered by 4 scales on each side; nuchals absent; loreals divided into 2 pairs; pre-oculars 2; presuboculars 1; supraciliaries 5, lower eyelid with 4 opaque scales; supralabials 6; a shallow groove on loreal-labial border, from lower corner of nasal across subocular obliquely downwards to the end of fourth supralabial; postocular 2; postsuboculars 3; primary temporals 3; secondary temporals 4; tympanum superficial, oval; mental bordered by postmental and first infralabials; postmental undivided; infralabials 6; three pairs of chin-shields; mid-body scales in 30 rows; 12 scale rows at position of tenth subcaudal on tail; dorsal scales subequal to ventral scales; scales on nuchal region keeled; vertebral scale rows keeled, but not widened, outer dorsal scale rows keeled; paravertebral scales 50; lateral scales strongly keeled, straight backwards; dorsal and lateral scales on tail distinctly keeled, two median keels on tail base not continuous on back; ventral scales smooth, in 44 transverse rows; 2 enlarged precloacals; postcloacal pores absent; subcaudals widened, about 2 times as broad as neighbouring scales, smooth, first one usually divided; scales on fore-limbs distinctly keeled, imbricate; those on hind-limbs keeled dorsally, smooth ventrally; subdigital lamellae smooth, numbering 14 under fourth finger and 19 under fourth toe.

**Colouration in life**. Dorsal surface of head, body and limbs brown with a few light spots; flank brown with light spots; venter white cream (Fig. 2c).

#### Distribution

In Vietnam, this species has been recorded from Cao Bang, Lai Chau and Thanh Hoa Provinces in the north southwards to Quang Ngai and Dak Lak Provinces (Dau et al. 2025). Elsewhere, the species is known from China (Uetz et al. 2025).

#### Ecology

The specimen was found at 21:00 h on a forest path. The surrounding habitat is secondary forest of medium and small hardwoods, mixed with shrubs and vines.

### Takydromus sexlineatus

Daudin, 1802

493DA1AB-A275-53FA-AD3C-2F10BF7BAF31

#### Materials

- **Type status:** Other material. **Occurrence:** catalogNumber: IB R.6499 (Field number TC-HN2025.34); individualCount: 1; sex: male; lifeStage: adult; occurrenceID: CBE278EA-B063-5E0C-A58B-7CCEE27F0130; **Taxon:** scientificNameID: *Takydromus sexlineatus*; scientificName: *Takydromus sexlineatus*; class: Reptilia; order: Squamata; family: Lacertidae; genus: Takydromus; specificEpithet: *sexlineatus*; scientificNameAuthorship: Daudin, 1802; **Location:** country: Vietnam; countryCode: VN; stateProvince: Ninh Binh; county: Kim Bang; municipality: Kim Bang; locality: Kim Bang SHCA; verbatimElevation: 60; verbatimLatitude: 20°32333"N; verbatimLongitude: 105°48415"E; verbatimCoordinateSystem: WGS84; **Event:** eventDate: 24 April 2025; eventRemarks: collected by Nguyen QT, Pham TC, Phan QT, and Pham VA; **Record Level:** language: en; collectionCode: Reptilia; basisOfRecord: Preserved Specimen

#### Description

Morphological characters of the specimen from Kim Bang agreed well with those in the descriptions by Smith (1935) and Taylor (1963): SVL 50.2 mm, TaL 172.9 mm; body slender with an extra-long tail; rostral large, pentagonal; supralabials 5; loreals 2; supraoculars 3; supraciliaries 3; frontonasal large; prefrontals 2, in contact with each other medially; a single frontal hexagonal; frontoparietals 2, in contact with each other medially; parietals 2, large, separated from each other; two occipital between two parietals; infralabials 6; temporal scales keeled; mental large; 3 pairs of chin-shields, two anterior pairs in contact with each other medially, three posterior pairs separated from each other by longitypinal gular scales; dorsal scales enlarged and keeled, ventral scales enlarged, keeled or smooth; scales on flanks small and granular; dorsal scales in 4 rows at mid-body; longitudinal dorsal scales 33; ventrals in 10 longitudinal rows; ventral scales 45; 2 rows of scales on lower flanks reduced, flattened, keeled; 7 rows of small granular scales on upper flanks; precloacal entire, enlarged, surrounded by a continuous series of moderate-sized scales anteriorly and laterally; one femoral pore on each side; 14 subdigital lamellae under fingers IV and 19 subdigital lamellae under toes IV.

**Colouration in life.** Dorsal surface of head and body brown, with two white stripes from the supraocular, extend through the outer-most row of dorsals and upper edge of hind-limb and ending at the posterior part of hind-limb; dorsal surface of tail and limbs brown; venter white (Fig. 2d).

#### Distribution

In Vietnam, this species has been recorded from Lao Cai, Cao Bang and Lang Son Provinces in the north southwards to Dong Nai and Lam Dong Provinces (Nguyen et al. 2009). Elsewhere, the species is known from India, China, Thailand, Laos, Cambodia, Myanmar, Malaysia and Indonesia (Uetz et al. 2025).

#### Ecology

The specimen was found at 10:15 h, on the ground near the residential area. The surrounding habitat is a vegetable garden with trees and shrubs.

### Ahaetulla prasina

(Reinhardt, 1827)

5418689B-C035-5C82-897D-FF36FFEF186A

#### Materials

- **Type status:** Other material. **Occurrence:** individualCount: 1; lifeStage: adult; occurrenceID: 716BB7EA-F415-5A22-9285-8451CB2A758B; **Taxon:** scientificNameID: *Ahaetulla prasina*; scientificName: *Ahaetulla prasina*; class: Reptilia; order: Squamata; family: Colubridae; genus: Ahaetulla; specificEpithet: *prasina*; scientificNameAuthorship: (Reinhardt, 1827); **Location:** country: Vietnam; countryCode: VN; stateProvince: Ninh Binh; county: Kim Bang; municipality: Kim Bang; locality: Kim Bang SHCA; verbatimElevation: 210; verbatimLatitude: 20°29121"N; verbatimLongitude: 105°50434"E; verbatimCoordinateSystem: WGS84; **Event:** eventDate: 26 July 2025; eventRemarks: observed by Pham VA and Bui VT; **Record Level:** language: en; collectionCode: Reptilia; basisOfRecord: photographs

#### Description

**Figure 3.**
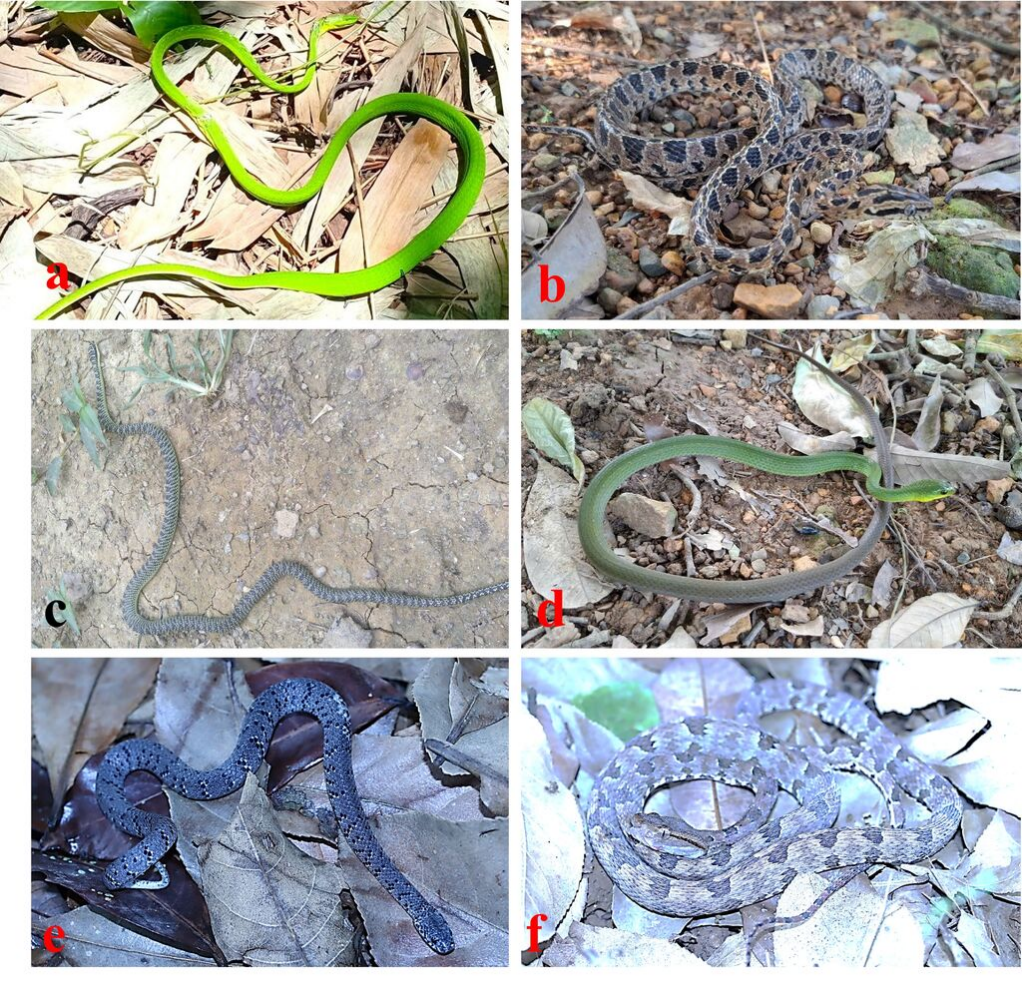
Snake species recorded in Kim Bang Proposed Species and Habitat Conservation Area, Vietnam: **(a)** *Ahaetulla prasina*; **(b)** *Boiga multomaculata*; **(c)** *Chrysopelea ornata*; **(d)** *Ptyas multicincta*; **(e)** *Pareas margaritophorus*; **(f)** *Protobothrops mucrosquamatus*.

Taxonomic identification was based on photographs taken on 26 July 2025. The body is very long and slender; head long, with a very long, pointed snout; dorsal surface green, with light spots on anterior part of body; a white or yellow line along the outer margin of the ventrals (determination after Smith (1943) and Hecht et al. (2013)) (Fig. 3a).

#### Distribution

In Vietnam, this species has been recorded from Lao Cai, Son La and Tuyen Quang Provinces in the north, southwards to An Giang and Ca Mau Provinces (Nguyen et al. 2009, Dau et al. 2025). Elsewhere, the species is known from China, Thailand, Laos, Cambodia, Singapore, Indonesia, Bangladesh, Bhutan, Brunei Darussalam, Hong Kong and the Philippines (Uetz et al. 2025).

#### Ecology

The specimen was observed at 15:42 h on a bush branch, near the forest path. The surrounding habitat is secondary forest of medium and small hardwoods, mixed with bamboo, shrubs and vines.

### Boiga multomaculata

(Boie, 1827)

DE5C4054-820E-5BB7-8411-24C8E0256F8F

#### Materials

- **Type status:** Other material. **Occurrence:** catalogNumber: IB R.6500 (Field number TC-HN 2025 223); individualCount: 1; lifeStage: adult; occurrenceID: BB90E424-B863-5E2B-87CA-6AAF3BD6B9AD; **Taxon:** scientificNameID: *Boiga multomaculata*; scientificName: *Boiga multomaculata*; class: Reptilia; order: Squamata; family: Colubridae; genus: Boiga; specificEpithet: *multomaculata*; scientificNameAuthorship: (Boie, 1827); **Location:** country: Vietnam; countryCode: VN; stateProvince: Ninh Binh; county: Kim Bang; municipality: Kim Bang; locality: Kim Bang SHCA; verbatimElevation: 280; verbatimLatitude: 20°29070"N; verbatimLongitude: 105°50591"E; verbatimCoordinateSystem: WGS84; **Event:** eventDate: 24 July 2025; eventRemarks: collected by Nguyen QT, Pham TC, Phan QT, and Pham VA; **Record Level:** language: en; collectionCode: Reptilia; basisOfRecord: Preserved Specimen

#### Description

Morphological characters of the specimen from Kim Bang were similar to those in the descriptions by Taylor (1965) and Kõhler et al. (2023): SVL 607.0 mm, TaL 158 mm; head distinct from neck; rostral as broad as high; internasal not in contact with loreal; prefrontal as long as half of frontal; nasal undivided; loreal 1/1, not in contact with orbit; pre-ocular 1/1; postoculars 2/2, bordering anterior temporals; anterior temporals 1/2, posterior temporals 2/2; supralabials 9/8, third and fifth touching the eye; infralabials 11/11, first to fourth bordering chin shields; dorsal scale rows 19–19–15, smooth, with vertebral scale row significantly enlarged; ventrals 213; cloacal plate entire; subcaudals 86, paired.

**Colouration in life.** Dorsal of head grey with three large dark stripes, a narrow line from eye across angle of mouth; small brown flecks on supralabials, edged in cream ventrally; infralabials cream, each with a small brown spot; dorsal surface brownish-grey, with two series of large brown spots, edged in cream; an irregular series of brown spots on outer edge of venter; chin and anterior part of throat cream-white; venter brownish-grey, with brown spots (Fig. 3b).

#### Distribution

This is a widespread species in Vietnam. Elsewhere, the species is known from India, China, Thailand, Laos, Cambodia, Myanmar, Malaysian Peninsula, Bangladesh, Hong Kong and Indonesia (Uetz et al. 2025).

#### Ecology

The specimen was found at 19:30 h, on the forest path. The surrounding habitat is secondary forest of medium and small hardwoods, mixed with shrubs and vines.

### Chrysopelea ornata

(Shaw, 1802)

686D8ACF-8C7B-59FF-8070-91AD156AC416

#### Materials

- **Type status:** Other material. **Occurrence:** individualCount: 1; lifeStage: adult; occurrenceID: 3ACAF4C9-9324-5584-A0AA-C63D5E6E9A70; **Taxon:** scientificNameID: *Chrysopelea ornata*; scientificName: *Chrysopelea ornata*; class: Reptilia; order: Squamata; family: Colubridae; genus: Chrysopelea; specificEpithet: *ornata*; scientificNameAuthorship: (Shaw, 1802); **Location:** country: Vietnam; countryCode: VN; stateProvince: Ninh Binh; county: Kim Bang; municipality: Kim Bang; locality: Kim Bang SHCA; verbatimElevation: 180; verbatimLatitude: 20°29479''N; verbatimLongitude: 105°49493"E; verbatimCoordinateSystem: WGS84; **Event:** eventDate: 28 July 2025; eventRemarks: observed by Pham VA and Bui VT; **Record Level:** language: en; collectionCode: Reptilia; basisOfRecord: Photographs

#### Description

Taxonomic identification was based on photographs taken on 28 July 2025. The body is slender; head distinct from neck; dorsal surface of head and body grey-blue, with bands in black and yellow on the head and black bands on the body; each light scale with a black median line (determination after Taylor (1965)) (Fig. 3c).

#### Distribution

This is a widespread species in Vietnam. Elsewhere, the species has been known from India, Nepal, Sri Lanka, Bangladesh, Myanmar, Thailand, West Malaysia, Laos, Cambodia, China and the Philippines (Nguyen et al. 2009 and Pham et al. 2020).

#### Ecology

One adult was observed at 14:10 h on the ground, near the forest path and one subadult was observed at 13:30 h on the ground, near the residential area. The surrounding habitat is secondary forest of medium and small hardwoods, mixed with bamboo, shrubs and vines.

### Ptyas multicincta

(Roux, 1907)

B54395D8-A435-58A0-8089-542C5ADC1C51

#### Materials

- **Type status:** Other material. **Occurrence:** catalogNumber: IB R. 6501 (Field number TC-HN 2025 230); individualCount: 1; lifeStage: adult; occurrenceID: 789E0E7B-F332-5044-8426-08BE5510176C; **Taxon:** scientificNameID: *Ptyas multicincta*; scientificName: *Ptyas multicincta*; class: Reptilia; order: Squamata; family: Colubridae; genus: Ptyas; specificEpithet: *multicincta*; scientificNameAuthorship: (Roux, 1907); **Location:** country: Vietnam; countryCode: VN; stateProvince: Ninh Binh; county: Kim Bang; municipality: Kim Bang; locality: Kim Bang SHCA; verbatimElevation: 260; verbatimLatitude: 20°28487N; verbatimLongitude: 105°50519"E; verbatimCoordinateSystem: WGS84; **Event:** eventDate: 24 July 2025; eventRemarks: collected by Nguyen QT, Pham TC, Phan QT, and Pham VA; **Record Level:** language: en; collectionCode: Reptilia; basisOfRecord: Preserved Specimen

#### Description

Morphological characters of the specimen from Kim Bang resemble those in the descriptions by Smith (1943) and Pham et al. (2014): body cylindrical; head moderately distinct from neck; eye large, pupil round; rostral as broad as high, partly visible from above; internasal not in contact with loreal; prefrontal as long as half of frontal; parietals longer than wide; nasal undivided; loreal 1/1, not in contact with orbit; pre-ocular 1/1; postoculars 2/2, bodering anterior temporals; anterior temporal 1/1, posterior temporals 2/2; supralabials 7/7, fourth and fifth touching the eye, sixth largest; infralabials 6/6, first to fourth bordering chin-shields; dorsal scale rows 15–15–15, smooth; ventrals 168; cloacal scale paired; subcaudals 99, paired.

**Colouration in life.** Dorsal surface green anteriorly, grey posteriorly, with whitish spots on body; belly whitish-green anteriorly, grey posteriorly (Fig. 3d).

#### Distribution

In Vietnam, this species has been recorded from Lai Chau and Lao Cai Provinces in the north southwards to Quang Ngai and Lam Dong Provinces (Pham et al. 2014). Elsewhere, this species is known from China and Laos (Uetz et al. 2025).

#### Ecology

Specimen was collected at 20:30 h on a tree branch, ca. 1.6 m above the forest floor. The surrounding habitat is secondary forest of small hardwood and shrubs.

### Pareas margaritophorus

(Jan, 1866)

51A9A441-0211-5EA5-8508-6CC5CD931632

#### Materials

- **Type status:** Other material. **Occurrence:** catalogNumber: IB R.6502 (Field number TC-HN 2025 89); individualCount: 1; lifeStage: adult; occurrenceID: 8CCCE962-E59F-5469-87EC-65C0812E50EA; **Taxon:** scientificNameID: *Pareas margaritophorus*; scientificName: *Pareas margaritophorus*; class: Reptilia; order: Squamata; family: Colubridae; genus: Pareas; specificEpithet: *margaritophorus*; scientificNameAuthorship: (Jan, 1866); **Location:** country: Vietnam; countryCode: VN; stateProvince: Ninh Binh; county: Kim Bang; municipality: Kim Bang; locality: Kim Bang SHCA; verbatimElevation: 210; verbatimLatitude: 20°31347"N; verbatimLongitude: 105°49'04"E; verbatimCoordinateSystem: WGS84; **Event:** eventDate: 24 April 2025; eventRemarks: collected by Nguyen QT, Pham TC, Phan QT, and Pham VA; **Record Level:** language: en; collectionCode: Reptilia; basisOfRecord: PreservedSpecimen

#### Description

Morphological characters of the specimen from Kim Bang were consistent with those in the descriptions by Smith (1943) and Hauser (2017): SVL 385.0 mm, TaL 65.0 mm; head distinct from neck; nasal undivided; loreal 1/1; pre-ocular 1/1; postoculars 1/1; subocular 1, long and slender, separating the eye from the labials; anterior temporal 1/1; posterior temporals 1/1; supralabials 7/7, third to fifth below the eye, seventh very long; infralabials 6/6; mental groove absent; dorsal scale rows 15–15–15, smooth; ventrals 145 (+ 1 preventral); cloacal scale undivided; subcaudals 36, paired.

**Colouration in life.** Grey-brown dorsally, with transverse bars on the back composed of black spots and small white dots; rudimentary brown on nuchal collar present; venter light brown with many dark blotches (Fig. 3e).

#### Distribution

In Vietnam, this species has been reported from Cao Bang, Hai Phong, Phu Tho and Quang Binh provinces (Nguyen et al. 2018). Elsewhere, the species is known from Malaysia, Indonesia, Thailand, Laos, Cambodia, Vietnam and China (Uetz et al. 2025).

#### Ecology

The specimen was found at 20:00 h on a tree branch, ca. 1.2 m above forest floor. The surrounding habitat is secondary forest of small hardwoods, mixed with shrubs and vines.

### Protobothrops mucrosquamatus

(Cantor, 1839)

1105F4D8-56A0-535C-85E2-CAA577256F18

#### Materials

- **Type status:** Other material. **Occurrence:** catalogNumber: IB R.6503 (Field number TC-HN 2025.106); individualCount: 1; sex: male; lifeStage: adult; occurrenceID: 7DB975E7-2FFB-5E1D-8419-0434A73B994A; **Taxon:** scientificNameID: *Protobothrops mucrosquamatus*; scientificName: *Protobothrops mucrosquamatus*; class: Reptilia; order: Squamata; family: Viperidae; genus: Protobothrops; specificEpithet: *mucrosquamatus*; scientificNameAuthorship: (Cantor, 1839); **Location:** country: Vietnam; countryCode: VN; stateProvince: Ninh Binh; county: Kim Bang; municipality: Kim Bang; locality: Kim Bang SHCA; verbatimElevation: 150; verbatimLatitude: 20°28444"N; verbatimLongitude: 105°50382"E; verbatimCoordinateSystem: WGS84; **Event:** eventDate: 25 April 2025; eventRemarks: collected by Nguyen QT, Pham TC, Phan QT, and Pham VA; **Record Level:** language: en; collectionCode: Reptilia; basisOfRecord: PreservedSpecimen

#### Description

Morphological characters of the specimen from Kim Bang were consistent with those in the description by Stuart and Heatwole (2008) and Pham et al. (2020): SVL 602 mm, TaL 150.3 mm; head triangular, clearly distinct from the neck; nasal undivided; internasals separated from each other by three scales; single loreal pit; two small scales between the nasal and the shield bordering the anterior region of the loreal pit; postoculars 2/2; supralabials 10/10, the third large, fourth separated from the subocular by two scales; temporals small; infralabials 14/15, the first pair in contact with each other, the first three pairs in contact with the chin-shields; dorsal scale rows 27-25-19, rhomboid, strongly keeled throughout, but smooth on the first outer row; ventrals 212 (+ 2 preventrals); cloacal scale undivided; subcaudals 84, paired.

**Colouration in life.** Dorsal surface of head brown, paler below; dorsal surface of body greyish-brown, with a series of large brown, dark-edged spots; a dark brown line extending from the eye to the angle of the mouth, edged in black; dorsal of tail light brown, with a series of conspicuous black spots; ventral surface brownish with white blotches (Fig. 3f).

#### Distribution

In Vietnam, this species has been reported from Lao Cai and Tuyen Quang Provinces in the north southwards to Quang Ngai and Gia Lai Provinces (Pham et al. 2020). Elsewhere, the species is known from India, Bangladesh, China, Taiwan and Myanmar (Uetz et al. 2025).

#### Ecology

Specimen was found at 21:30 h on the forest path. The surrounding habitat is secondary forest, composed of medium and small hardwoods, liana and shrub.

## Discussion

Our new records bring the known reptile diversity of the Kim Bang SHCA to 27 species, comprising two species of the family Agamidae, five species of the family Gekkonidae, one species of the family Lacertidae, one species of the family Scincidae, one species of the family Pythonidae, nine species of the family Colubridae, one species of the family Homalopsidae, three species of the family Elapidae, one species of the family Viperidae and one species of the family Geoemydidae (Do et al. 2022). Amongst new distribution records of lizards, *Gekko palmatus* is a widely distributed species from all sites in Kim Bang SHCA, in a residential area, disturbed secondary and evergreen forest. Whereas other species were recorded only at one site, for example, *Hemidactylus garnotii* was found in the forest near Tam Chuc Pagoda, *Tropidophorus hainanus* was found in the forest near Lien Son Quarry and *Takydromus sexlineatus* was found in the forest near Ba Sao Golf Course. Amongst new distribution records of snakes, *Ahaetulla prasina* was found from the forest near Lien Son Quarry, *Boiga multomaculata* from the forest near Ba Bac Quarry, *Chrysopelea ornata* from the forest near Tam Chuc Pagoda and Ba Bac Quarry, *Ptyas multicincta* from the forest near Lien Son and Ba Bac quarries, *Pareas margaritophorus* from the forest near Ba Sao Golf Course and *Protobothrops mucrosquamatus* from near the forest Dong Tam Quarry.

The discovery of new distributional records of reptile species in limestone karsts further highlights the importance of these ecosystems in generating and harbouring endemic diversity. Although northern Vietnam has one of the most extensive limestone karst systems in the region, it remains insufficiently understood (Clements et al. 2006). Recent surveys, for example, Do et al. (2020), Luu et al. (2020), Nguyen et al. (2020), Ha et al. (2022), Luu et al. (2023), Luu et al. (2024), Pham et al. (2024), Luu et al. (2025) and Bragin et al. (2025)

continue to discover new species of reptiles from the region. However, it is likely that additional new species in the genus will be found if more expeditions are conducted in the karst system.

Currently, only 27 species have been recorded in the Kim Bang SHCA and this number is expected to increase with future surveys. Compared to neighbouring areas, the number of recorded species is much lower, for example, 51 species have been recorded in Thuong Tien Nature Reserve, 63 species in Pu Luong Nature Reserve, 50 species in Ba Vi National Park and 63 species in Xuan Nha Nature Reserve (Luu 2011, Luu et al. 2020, Pham et al. 2022 and Dau et al. 2025).

## Supplementary Material

XML Treatment for Gekko palmatus

XML Treatment for Hemidactylus garnotii

XML Treatment for Tropidophorus hainanus

XML Treatment for Takydromus sexlineatus

XML Treatment for Ahaetulla prasina

XML Treatment for Boiga multomaculata

XML Treatment for Chrysopelea ornata

XML Treatment for Ptyas multicincta

XML Treatment for Pareas margaritophorus

XML Treatment for Protobothrops mucrosquamatus
